# ‘Testing Together Challenges the Relationship’: Consequences of HIV Testing as a Couple in a High HIV Prevalence Setting in Rural South Africa

**DOI:** 10.1371/journal.pone.0066390

**Published:** 2013-06-18

**Authors:** Hanani Tabana, Tanya Doherty, Birgitta Rubenson, Debra Jackson, Anna Mia Ekström, Anna Thorson

**Affiliations:** 1 Health Systems Research Unit, Medical Research Council of South Africa, Cape Town, South Africa; 2 Department of Public Health Sciences, Karolinska Institutet, Stockholm, Sweden; 3 Department of infectious Diseases, Karolinska University Hospital, Stockholm, Sweden; 4 School of Public Health, University of the Western Cape, Bellville, Cape Town, South Africa; Rollins School of Public Health, Emory University, United States of America

## Abstract

**Objective:**

We conducted qualitative individual and combined interviews with couples to explore their experiences since the time of taking an HIV test and receiving the test result together, as part of a home-based HIV counselling and testing intervention.

**Methods:**

This study was conducted in October 2011 in rural KwaZulu-Natal, South Africa, about 2 years after couples tested and received results together. Fourteen couples were purposively sampled: discordant, concordant negative and concordant positive couples.

**Findings:**

Learning about each other’s status together challenged relationships of the couples in different ways depending on HIV status and gender. The mutual information confirmed suspected infidelity that had not been discussed before. Negative women in discordant partnerships remained with their positive partner due to social pressure and struggled to maintain their HIV negative status. Most of the couple relationships were characterized by silence and mistrust. Knowledge of sero-status also led to loss of sexual intimacy in some couples especially the discordant. For most men in concordant negative couples, knowledge of status was an awakening of the importance of fidelity and an opportunity for behaviour change, while for concordant positive and discordant couples, it was seen as proof of infidelity. Although positive HIV status was perceived as confirmation of infidelity, couples continued their relationship and offered some support for each other, living and managing life together. Sexual life in these couples was characterized by conflict and sometimes violence. In the concordant negative couples, trust was enhanced and behaviour change was promised.

**Conclusions:**

Findings suggest that testing together as couples challenged relationships in both negative and positive ways. Further, knowledge of HIV status indicated potential to influence behaviour change especially among concordant negatives. In the discordant and concordant positive couples, traditional gender roles exposed women’s vulnerability and their lack of decision-making power.

## Introduction

Southern Africa remains the region most affected by the HIV epidemic with 31% of global new infections and 34% of global AIDS deaths despite the dramatic decrease in HIV incidence in most countries in the region in 2011 [1]. In this hyper endemic context, transmission of HIV occurs primarily through heterosexual intercourse, with a large proportion of new HIV infections occurring in discordant cohabiting couples, many of whom are unaware of each other’s sero-status [2], [3]. South Africa continues to have the largest number of people infected with HIV in the world [4]. The HIV epidemic is generalized and has stabilized for the past four years at an antenatal prevalence of 30% [5].

A survey undertaken in 2010 found that 60% of adults in South Africa knew their HIV status [6]. In an effort to address the high HIV prevalence, the South African government launched a national HIV counselling and testing (HCT) campaign in 2010, targeting 15 million South Africans of which 25% of the total population took a test for HIV by June 2011 [7].

The high HIV infection rates attributable to heterosexual transmission in sub-Saharan Africa have led to increasing efforts to evaluate the extent of HIV transmission within marriages or cohabiting partnerships [8], [9]. This has led to the recognition of couple HCT as a strategy to improve testing rates and a gateway to prevention and treatment [10], [11]. However, still very few couples in high prevalence areas have been tested together and barriers to couple HCT have been documented [12]. Couple HCT has the potential to improve use of HIV prevention strategies when both partners test together and know their HIV status as it presents opportunities to discuss concordance, and discordance and consequences thereof. The HIV status guides the type of counseling, and that has implications for the next steps that the couple has to take, for example accessing treatment or health care needed. Further, couple HCT facilitates the identification of discordant couples eligible for treatment as prevention (TASP) [13].

Larsson et al. (2009) conducted a study in Uganda that explored men’s views on and experiences of couple HCT during antenatal care (ANC). They found that men were aware that couple HCT was available but the study highlighted a number of barriers to uptake, such as health worker attitudes, unstable and distrustful marriages, and fear of conflicts with their partners [10].

The Rwandan model of couple HCT [14], which promotes male involvement and encourages HIV disclosure, provides a supportive environment that facilitates management of sero-discordant results, especially during pregnancy. HCT within an ANC context is an entry point for prevention strategies related to HIV transmission during pregnancy, such as encouragement of consistent condom use and the availability of antiretroviral therapy (ART) for eligible HIV positive male partners [12], [15]. However, barriers to couple HCT still exist due to fear of abandonment, rejection and discrimination, violence, upsetting family members, and accusations of infidelity [9], [16]. In an effort to address these challenges, the World Health Organization has released new guidelines on couple HCT and see it as a priority [17].

A number of studies have explored couple HCT to prevent HIV/AIDS transmission in settings with high HIV prevalence. However, few studies have examined the psychosocial impact of couple counselling and testing on the couple’s relationship [18], [19]. Rispel et al. (2012) in their exploration of experiences of living with HIV have studied social and relational challenges including gender dynamics, sexual relationships and reproductive decision making among discordant couples in South Africa [20], [21]. In their study, they found that, discordant couples where partners tested separately and later disclosed had to deal with the emotional and sexual impact of HIV discordance on the couple relationship, reconciling the desire for children with preventing transmission of HIV to the negative partner, disclosure of the HIV infection to friends, families and others, and well-being of the HIV positive partner. Research on social situations of couples living with HIV/AIDS needs to include discordant as well as concordant positive and negative couples to further understand how the HIV status affects the couple relationships.

Undergoing HCT together and receiving test results at the same time should avoid delayed disclosure, delayed access to care and treatment and other opportunities that couple HCT introduces. The aim of this study was to explore the experiences of couples after undergoing home-based couple HCT together and receiving the test result together in rural KwaZulu-Natal province in South Africa, a province with the country’s highest HIV prevalence of 15.8% [22].

## Methods

This qualitative explorative study was conducted within a cluster randomized controlled trial (Good Start) that aimed to assess the effect of home-based HCT in rural KwaZulu-Natal province in South Africa. The intervention was door-to-door HIV counselling and testing for all consenting adults aged 18 years and above and youth, 14–17 years with parental or guardian consent. In homes where there were couples, these were offered HCT together; they were counselled together and received test results together. The intervention was delivered by trained lay counsellors and included counselling and testing, HIV/AIDS education including HIV risk reduction. For this qualitative study, only couples who were counselled and tested together, received their HIV test results together and agreed to be part of the study were included as the aim was to explore couple relationship experiences since time of testing. There are several studies that have looked at couples testing separately and disclosing but not mutual testing and sharing of results. Full details of the intervention are described elsewhere [23].

### Ethics Statement

The randomized control trial received ethical approval from the South African Medical Research Council and the KwaZulu-Natal provincial research committee. Ethical approval for this qualitative study was received from the South African Medical Research Council.

Written informed consent was obtained from each person separately prior to participation. All information sheets and consent forms were translated into the local language and back translated into English by the first author to ensure correct use of language. After hearing the first author read the information sheet aloud, the potential participant was asked to report back a description of the expectations in his or her own words and explain the reasons why they were prepared to participate in the interview. The same procedure was followed with the consent form. This was done to assist the interviewer in determining whether participants fully understood the contents. Individuals were provided with information on how to contact the study staff to report adverse events associated with the interview process. Participants were informed that they could withdraw from the interviews at any time without giving any reasons and without consequences for them.

### Setting

The study community, Umzimkhulu sub-district, is located in KwaZulu-Natal province, Sisonke district in South Africa. This is one of the poorest rural areas in South Africa where 77% of households live below the poverty line with poor access to basic services like electricity, piped water, and toilets [24]. Sisonke has approximately 304 000 people. The district has seven hospitals, and about 30 clinics that offer HCT. The antenatal HIV prevalence in Sisonke district was 39.9% in 2011 [5] and a baseline survey conducted in Umzimkhulu sub-district in 2008 found that only 32% of adult men and women had ever had an HIV test [25].

In this area, agriculture is the main activity, which includes cattle ranching, sheep, goats, pigs and crop cultivation. A smaller part of the population has informal employment, and some are migrant labourers. Men leave their partners for work in urban areas, mostly in the mining sector [26] and return home periodically especially during public holidays, such as Christmas. The system of migrant labour dates back to the apartheid era when movement of South Africa’s black population was controlled to maintain a steady supply of labour. Circular migration has been reported to exacerbate the high HIV prevalence levels in rural KwaZulu-Natal [27].

Polygamy is part of the culture and associated with manliness, where having multiple concurrent sexual partners is a celebrated norm [28]. Historically in rural KwaZulu-Natal it was socially acceptable or, a ‘right’ for men and women (unmarried), to have more than one courting partner. However, transitions of this norm led to the emergence of multiple partnering idealizing masculinity for men, while women were expected to remain monogamous [28].

Women generally lack influence in society. Most men assume the head of household role and these hierarchies form the gender order of the society. Women are often unable to freely express themselves and are overpowered by men. The gendered manner of these relationships also perpetuate in relationships of sexual or intimate nature [29].

### Participants

Couples who participated in the Good Start home-based HIV counselling and testing (HBHCT) intervention were asked if they were prepared to participate in a follow up research project. For this study, a couple was defined as concordant negative when both partners were HIV negative, and concordant positive when both were HIV infected. A discordant couple is one in which only one partner was HIV infected.

Among those who agreed and signed the informed consent form, heterosexual couples were purposively selected to include concordant positive, concordant negative and discordant couples. Another criterion for participation was that the couple should be willing to discuss openly and honestly about their experiences of counselling and testing together and their life together after testing. After having interviewed 14 couples, there was no new information forthcoming. When looking for younger couples (where both partners were age 24 years or younger), it was found that they had already separated or left the village and in one instance one partner had died thus our participants were aged 25 or older. Characteristics of the couples are presented in Table 1.

**Table 1:** Couple characteristics.

Couple	Gender, Age	Number of children	Economic status (income, living benefits,child grants)	HIV status
1	M, 49 F, 44	–	Both man and woman subsistence farmers	Concordant negative
2	M, 62 F, 59	4 children	Both man and woman subsistence farmers	Concordant negative
3	M, 41 F, 35	4 children (ages 4–11 yrs)]	Child grant, man gets government disability grant	Concordant negative
4	M, 68 F, 40	3 children, 2 in school +1 disabled	Child grant+child disability grant, both man and woman subsistence farmers	Concordant negative
5	M, 50 F, 32	4 children (1 school, 2 nursery,1 toddler)	Child grant, both man and woman subsistence farmers	Discordant M (+), F (−)
6	M, 34 F, 37	4 children	Child grant, man had a piece job, both man and woman subsistence farmers	Concordant positive
7	M, 61 F, 57	1 school child	Child grant, man works on piece jobs, both manand woman subsistence farmers	Discordant M (+), F (−)
8	M, 57 F, 56	4 school going children (6–16 yrs)	Both man and woman subsistence farmers	Concordant positive
9	M, 33 F, 30	4 children, 2 school, 2 nursery school	Child grant, man lost job due to illness, both manand woman subsistence farmers	Discordant M (+), F (−)
10	M, 62 F, 54	2 children	Man gets pension, both man and womansubsistence farmers	Discordant M (+), F (−)
11	M, 59 F, 48	7 children (incl. grand children)	Man gets pension (R1000/month)	Concordant positive
12	M, 38 F, 23	2 children (1 school, 1 toddler)	Child grant, both man and woman subsistence farmers	Concordant positive
13	M, 33 F, 33	4 children	Man gets piece jobs, subsistence farming	Concordant positive
14	M, 65 F, 46	2 children (3+4 yrs)	Both man and woman subsistence farmers,man also gets pension	Discordant M (−), F (+)
*15	M, 24 F, 21	Information not available		Discordant M (−), F (+)
*16	M, 23 F, 18	Information not available		Discordant M (+), F (−)
*17	M, 22 F, 16	Information not available		Discordant M (−), F (+)
*18	M, 20 F, 19	Information not available		Discordant M (−), F (+)

**Note:** M = Male, F = Female, (+) = HIV positive partner, (−) = HIV negative partner.

* These couples were not interviewed as they had separated at the time of conducting interviews.

Formal employment is scarce in this community. A few men received a government social security grant or had what is locally referred to as ‘piece jobs’ (unstable, insecure and temporary employment lasting only a few days) at the time of the interviews. However, most men had had some form of employment as migrant labourers in urban areas and had returned home mostly due to sickness or some form of disability. Most of these couples survived on the government sponsored child support grant (about US$33 per month for children between 0–18 years from poor families).

### Data Collection

The first author and interviewer (HT) is a young black South African woman sharing ethnicity and language with the participants, and residing in an urban area outside the community. An assistant researcher, originating from the study sub-district made introductions in all households. The first author interviewed couples face-to-face on testing together as a couple during the home-based HCT intervention. The couple interviews were conducted in October 2011, which was approximately 1–2 years following home-based testing. All interviews were performed in a private place of their choice first together and then individually in privacy on the same occasion. We decided to interview the couples both together and then individually to get different perspectives. An interview guide with open-ended questions was used. All interviews were conducted in the local language (IsiXhosa/IsiZulu). Questions asked in the combined interviews were repeated again in the individual interviews to allow the person to discuss anything they were not able to raise in the combined interview. At the end of the interview, the participants were asked what recommendations they would have for other couples with regards to couple HCT. Probing was done where needed to gather greater depth of information. Information saturation was reached after 3–4 couples in each group (discordant, concordant negative and concordant positive) as each group was interviewed extensively to get rich information. To ensure saturation, another couple in each group was added. The interviews were audiotaped and took between 20–60 minutes.

### Analysis

The audiotapes were transcribed verbatim and translated to English then back translated to the local language (Zulu/Xhosa). The first author who is fluent in all three languages checked all the translations by repeatedly listening to the audiotapes and making sure that all conversations had been captured, and no meaning lost in the process of the translation.

The first author (HT) repeatedly read the transcripts to understand and identify the meaning of the interviews. Co-authors BR and AT also read the transcripts. The data was analysed using latent content analysis [30]. Meaning units were identified, coded and grouped into categories by the authors (HT, BR and AT). Similar categories were merged together and sub-themes and a main theme were developed. In cases where there were disagreements in themes or meanings of data, the three authors discussed until a consensus was reached. Although couples were interviewed combined, then separately, there were no instances of discrepancies in what was reported in both interviews. Quotes are used to illustrate the informants’ views. Couple numbers as depicted in table 1 will be used throughout the text to refer to informants.

## Findings

In the analysis the main theme, ‘*testing together challenges the relationship*’ was developed based on the sub-themes: ‘*Knowledge (of HIV status) is empowering?*’ ‘*Intimacy lost and found? and ‘To trust or mistrust?*. There was evidence that supports but also contradicts positive outcomes and experiences of the testing, which we attempt at reflecting in the ambiguity of the sub-themes. The testing experience is evidently not placed in a neutral ground, but is highly dependent on prevailing gender structures, which e.g. seemed to impair the potential of empowerment for women. In all discordant couples but one, men were the positive partners.

### Knowledge (of HIV status) is Empowering?

Knowledge of HIV status had different consequences for the couples depending on their HIV status and gender. The phenomenon of empowerment through knowledge is widely understood and accepted. However knowledge is not enough to empower people. In this study knowledge helped both men and women to realize the need to come to terms with their status and the need to change behaviour. For both men and women the social norms and traditions made it difficult to use the knowledge especially for women who lacked the power to act on their new knowledge. By learning about their partner’s status the women gained some courage to question their husband’s behaviour or to require respect for their own negative status. Due to the deeply rooted gender imbalance the men continued to get sex when they wanted even if it meant this was against the woman’s will.

These women’s immediate reactions to their partner’s positive status included anger, hurt and even separation where women went back home to their parents’ households. They however, came back to their partners after persuasion by their in-laws, or because of fear of being gossiped about by neighbours. Couples found ways to continue life together, as partners in their situations.

…. He would come and look for us and apologize and I would come back and his family would come as well and they would apologize as well on his behalf and beg me to stay. I get tired living with someone who doesn’t even support his household because he doesn’t even give me money, because now I live off the children’s grants, before that life was very difficult for us. ***(*** *Couple 5, discordant, individual interview with woman* ***).***

… I was afraid that if I stayed a long time at home people were going to start gossiping about me and say I left my husband because he was sick. That is why I came back. **(** *Couple 12, concordant positive, individual interview with woman* **)**

…. We moved on with life, and accepted, and now and again he reminds me to take my pills…. Yes, he supports me a lot. When I go to fetch my medication from the clinic, he usually accompanies me. (*Couple 14, discordant, individual interview with woman*)

Men especially in concordant negative couples were empowered by knowledge of their HIV status and it made them consider abstaining from risk behaviour. Testing seemed to have been an awakening of the importance of fidelity as men expressed wanting to end any extra sexual relationships while for their partners, a concordant negative status meant women were willing to start afresh and overlook infidelity. Men were relieved and for them this was an incentive for fidelity and change of behaviour.

…After the counsellor tested us, we were relieved and then I thought if I was doing something on the side then I would have made up my mind to stop now that I know my status. *(Couple 3, concordant negative, combined interview, man’s comment)*

For women in concordant positive relationships knowledge of status made their partners more available in their lives, a reduction in violent acts and possibly an end to infidelity. Men usually decide what relationships they want and when to have an extra sexual partner, while women hope their men do not have other partners.

It is better now *(referring to relationship with husband*) it means he has stopped sleeping outside (with other women). …. It’s not the same as before. Also, he used to beat me up for small things when we fought. Also I used to keep quiet about my feelings but now I say whatever I feel … He doesn’t hit me now because he just feels guilty, he knows very well that he is the one who caused this whole thing (bringing HIV into the relationship). **(** *Couple 11, concordant positive, individual interview with woman* **)**

Some couples suspected they were HIV infected but never sought HCT services due to fear of stigma and being gossiped about. Knowledge of HIV status empowered couples to overcome their fears and access the care they need.

…. We had seen something wasn’t adding up, we were suspecting we might have it. So we just thought we should get tested because they (counsellors) arrived here when we were thinking of doing it because we don’t want people gossiping about us being sick….Now we have tested and know what to do (accessing care), so we don’t care what people say about us anymore. *(Couple 6, concordant positive, combined interview, woman’s comment)*

In this high HIV prevalence setting, deaths related to AIDS are common and being sick naturally raises suspicions of HIV infection. This motivated some people to want to know their status.

…When you get sick these days you will be labeled as having the disease, so testing made us know where we stand and relieved us. One can’t even get sick because my wife will suspect me of having it. *(Couple 2, concordant negative, individual interview with man)*

On the other hand, in a context where ARV treatment is widely available, to be aware of one’s HIV status could also imply an opportunity to take control of the situation instead of thinking of HIV infection as a death sentence.

…. During my test it came to light that she should test as well so that we know both our status and can then protect ourselves by accessing the care we need, that is how I asked her in and encouraged her that we test together. It is good to know one’s status because sometimes you might be thinking you are fine and yet you are infected, and so if the sickness is there, then it is best to know as soon as possible and rectify the problem. *(Couple 9, discordant, individual interview with man)*

### Intimacy Lost and Found?

For older couples, 40 years and above (both husband and wife), abstinence and loss of sexual intimacy was not so difficult, while for younger couples the risk of infection or reinfection was a threat that became difficult to handle. Women in discordant couples (with a positive husband) tried to avoid sexual intimacy, but with the risk of violence in the form of sexual coercion, physical abuse or threats of being left for a girlfriend.

Ever since we found out about his status, I just don’t feel like having sex with him. Sometimes when he asks me, I tell him that I’m tired and sometimes tell him that I don’t want to have sex with him… Sometimes he pushes me away and says that the reason I’m like this is because of his status, and I would say I just don’t have any feelings…I do it when I don’t want to sometimes. *(Couple 5, discordant, individual interview with woman)*

Testing together also provided opportunities for renewed intimacy (closeness) and strengthened affection. For concordant negative couples it meant renewed promises of fidelity.

…. There was a huge difference; my wife was now warm and attentive. She also can see a difference but we’ve never really discussed it. By the time she (counsellor) left, we knew our status and knew that now we have to maintain it this way until we die. This also made a difference in our love for each other, and we still have it. It was nice to see that we have both kept ourselves free from the virus. *(Couple 3, concordant negative, individual interview with man)*

For concordant positive couples testing together created opportunities for closeness as they saw their HIV infection as something they were into together. There was a ‘*we-ness’* expressed in their narratives, they had processed emotions and the cost that accompanied a HIV positive status, and status knowledge ascertained suspicions of being infected.

**Woman: ….**I was relieved young lady because we found out something we suspected that we had, we knew there was a possibility of us having it….So we had the results and we saw that we needed help. So we were happy when we saw people from Good Start. They were a huge help I don’t want to lie. There was a change between us because we had differences we were arguing about. This thing made us sit down and talk things through. No shame everything is now good, we live for each other. We are very happy. **(** *Couple 6, concordant positive, combined interview, woman’s comment* **).…**I decided I’m not going to withhold my affections from him. This has made our love even stronger. The counsellor told us what to do in order to carry on with our lives. **(** *Individual interview* **)**

**Man: ….**I would like to add on what my wife had already said. We do have our differences sometimes but we also see that what we are doing is not going to get us anywhere. Because we know we need to support each other so we can carry on with our lives, and be there for each other. Our relationship is very good, and she supports and encourages me most of the time. She is the one who has made me accept my status. She tries and builds me up when I'm feeling down. She counsels me and supports me, and she doesn’t blame me for anything. **(** *Couple 6, combined interview, man’s comment* **)**

### To Trust or Mistrust?

Fidelity is preached as a preventive method and seen as an *insurance* against HIV infection but is a recognised challenge worldwide. The couples agreed to test together thereby also accepting the risk of receiving devastating information about themselves as well as their partners. According to the women, despite discordant results, discussions about the men’s infidelity were unnecessary. The positive status became proof of infidelity and a negative result a sign of one’s innocence.

In a culture where multiple sexual partnerships are common reasons for mistrusting one’s partner are constantly present. Testing together meant for some couples, in this case mostly women, that their suspicions could be confirmed. Concordant negatives regained trust with their newly found status.

…I saw it as a way to regain our trust because in as much as we never mentioned this, there was mistrust, and so it brought relief, and renewed our trust. We might not have been saying it but we didn’t trust each other. It is good, very good because you feel free after you have tested *(couple 2, concordant negative, combined interview, man’s comment)*

…. I suspect he had sexual partners here in the village. Our test results would have been the same if he did not have other sexual relations but they were different. I did not need to ask him about his extra sexual partners, those questions were not needed as the results speak for themselves *(couple 10, discordant, individual interview with woman)*

Men had knowledge that MSP was associated with a risk of acquiring HIV/AIDS, but nonetheless engaged in it. MSP was described to have been the norm ‘*back in the days’* hence condoned and encouraged for men. However, these men stated that the risk of disease (HIV) requires caution and thus makes MSP less viable as a sexual behavior.

…Being unfaithful was fine back then. It was fine for men to have as many women as they wanted. There were not so many diseases around, but nowadays you must look after yourself. *(Couple 1, concordant negative, individual interview with man)*

## Discussion

To our knowledge, this is the first study to explore experiences of couples after having tested together at home, and living together with mutual knowledge of their HIV status. Couples interviewed had remained together for at least two years since taking a home-based HIV test at the same time. Thus they had adjusted to the knowledge and found ways to cope with their different statuses.

We found that testing together affected relationships both positively and negatively. Mutual status knowledge forced couples to face their situation and find ways forward within their social and emotional situations. Our findings highlight the couples’ ability to act on knowledge, while illuminating women’s (for those in concordant positive and discordant partnerships) lack of power to act in a way that protected them.

Couple testing benefitted concordant negative couples where the men discussed the need for behaviour change including ending extra sexual relationships. Discordant couples faced more challenges than concordant couples. In discordant couples, the negative partner usually the woman was faced with the challenge of maintaining an HIV negative status in a relationship that was characterized by male dominance. As a consequence, women refused to engage in sexual activities due to fear of infection, a finding that has been reported by others previously [31], [32]. For both men and women this was difficult to handle, and often led to subordination, IPV or that the man mentioned finding other sexual partners to satisfy his desire, which would increase the woman’s vulnerability.

Where norms include male control over sexual decision-making, it is uncommon for women to refuse sex and when this happens it may introduce or exacerbate IPV [33]. Our findings confirm previous reports of occurrences of IPV (in the form of sexual coercion) [33] and loss of sexual intimacy [20], [31], [32] as a consequence of a positive HIV test result among couples. After two years of living together with mutual knowledge of status, these negative consequences still prevailed in the relationships without concrete ways of coping with the situation. Therefore a programmatic challenge is assisting couples to find solutions, especially for discordant couples, on how to cope with knowledge of HIV status and the ability to develop a mutual agreement to continue with their sexual life while maintaining a negative status for the un-infected partner. Also, ensuring long-term protection against HIV infection in a context where MSP is a norm is a challenge. These couples need to be empowered to live with the positive status and take ARV treatment correctly to also benefit the negative partner.

Women’s lack of self-assertiveness in a gendered, male dominated society leads to a lack of ability to protect themselves from the risk of infection. There is also lack of support structures to help women deal with such situations, and the society does not support women who challenge men on sexual matters. In most instances when infidelity was suspected, there was no confrontation of the issue, while for women who had the courage to ask their partners, the man still could deny or admit with no resolve on the issue. This is expected in this context where men often dominate relationships and take the liberty to enjoy extra relationships without being answerable to their intimate partners [28]. Some men in this study discussed willingness to end extra sexual relationships demonstrating their awareness that MSP can have negative consequences, such as increased risks of HIV transmission. These gender norms where high-risk behaviour is encouraged among men, also increase men’s vulnerability to HIV [1].

While most of the couple relationships were characterised by mistrust, loss of sexual intimacy and living with uncertainty about their partner, concordant negative couples regained trust after testing. Regain of trust was also perceived to be a benefit of couple HCT in a formative study to assess acceptability of a HBHCT intervention to improve HCT uptake and disclosure in rural Tanzania [16]. The reported reactions among the couples testing positively or being discordant, revealed beliefs of erroneous interpretations of HIV transmission, such as the fact that HIV infection had come post-marriage (as opposed to before), or that sexual interaction with an HIV positive individual would always lead to transmission. HIV transmission is a complex issue, whether treatment is available or not, and this needs to be better addressed and communicated in couples’ testing programmes for these to draw on all potential benefits of shared knowledge for the couple. Issues of trust due to newly diagnosed HIV infection and how to cope with the negative consequences of this knowledge are challenges that have been previously reported on experiences of both homosexual [34] and heterosexual couples [18], [35].Ways of making couple HCT an attractive approach are urgently needed, and these need to be context specific to address the social factors in a given society.

One way of handling the challenge of unfavourable outcomes of testing together can be to separate, which was the choice of the younger discordant couples tested together. For the older couples, this solution was not approved by society and most women chose or felt obliged to return to their partners. Women feared being criticised, judged and gossiped about for leaving the marriage. In rural KwaZulu-Natal being married is a dignifying ‘status’ for men and women. Women were probably pressured by pride placed in marriage, regardless of the strife caused by the newly found HIV status. The societal expectation that a woman has to remain strong in a marriage and endure the hardships that come with it pressured women to return [36].

In three of the four young couples, who were not available for interviewing, it was the woman who was the positive partner (Table 1). Plausible reasons for their separation could be blame, violence, fighting and abandonment. Women continue to bear the greater vulnerability to HIV due to their dependency on men, lower socioeconomic status and fear of violence and hence have little or no power to take steps to protect themselves [1].

In our study, participants appreciated being offered a test at home as lack of initiative, fear of stigma, and fear of lack of confidentiality had hindered them from taking the test. After learning their status, couples needing care or treatment took initiative to go to the clinic. This highlights the benefit of HBHCT in reaching ‘hard to reach’ populations that otherwise would not have initiated accessing HCT services.

These attitudes have implications for the wide spread roll-out of ARVs in South Africa if those eligible are not presenting for care. Moreover, the possibility to reduce within couple transmission among discordant couples by early treatment initiatives [37] is completely lost.

The new WHO guidelines on couple HCT are timely as there is an urgent need to continue to explore strategies to increase HCT especially among couples in high HIV prevalence settings. Encouragingly, the guidelines recommend offering ART for the HIV positive individual in a sero-discordant couple even when he or she is not eligible for ART according to the current guidelines of a CD4 cell count <350 as a means of reducing HIV transmission [17]. Lately there has been increased recognition of the importance of couple HCT yet still few couples seek these services. In future, couples should be provided with options to deal with the challenges that couple testing creates. Interventions need to hone in on addressing gender imbalances and challenge societal norms. Further, couple counselling should address misconceptions about the meanings of HIV infection attributed to unfaithfulness to avoid issues of blame as this might act as a barrier to couple testing or disclosing of results. Women presumed HIV infection was the result of unfaithfulness of their male partner- an unproven speculation though these men admitted to it. Men probably reasoned the same way given that some discordant couples where the woman was positive had separated by the time interviews were conducted.

### Methodological Considerations

The findings in this study represent what the participants shared about having tested together two years after the test. It gives information about the experiences of how couples managed their lives together after the mutual knowledge about their status. Views and attitudes to testing together among those who decided not to take the test will be different and needs further study. During interviews some participants did speak openly about their situations probably due to the sensitive nature of the issues discussed.

Furthermore, the four youngest couples had all separated and could not be interviewed, as they were not available as a couple. These younger couples did not have the same motivations as older couples to remain in their relationships. These were the discordant couples with a positive woman; unfortunately no discordant couple where the woman is positive was available for interviewing.

The findings in this study refer to a local community in rural South Africa, but the findings relate to phenomena common in many societies such as gender imbalances outweighing knowledge about needed behaviour change, the difficulty of upholding fidelity and ability to trust each other in a context of accepted (by men for men) multi-partnering and the difficulties in managing sexual desire and intimacy in a discordant couple. Thus the findings can be relevant in many settings with similar social constructions.

### Conclusions

These findings highlight the positive effects that HIV status knowledge has on initiating behaviour change, in concordant negative couples, while also illuminating the impact of cemented gender roles on women’s vulnerability in the discordant couples. Couples had to respond to the challenges that testing together brought to their relationships. Couples did not take initiative to go to the clinic to access HCT services prior to home-based HCT.

Future interventions should address the social consequences of knowledge of HIV status within couples and give guidelines on how to live with HIV, and to cope with discordance in a relationship. Given the marked power-imbalance in discordant couples, where the woman is HIV negative, sexual coercion by a known HIV positive husband is day-to-day life. Actions to increase early treatment start will have immense implications in terms of reducing the actual transmission risk for the women and home-based couple testing could be one way to reach this goal. Structural interventions to increase gender equity, improve trust, partner communication and other couple relationship dynamics such as sexual intimacy are urgently needed. Further, couple counselling should address misconceptions about the meanings of HIV infection attributed to unfaithfulness to avoid issues of blame as this might act as a barrier to couple testing or disclosing of results.

Addressing the above-mentioned issues would build a stronger theoretical and methodological basis for couple oriented HIV prevention in order to make couple HCT an attractive model.
